# Prefoldin subunits (PFDN1–6) serve as poor prognostic markers in gastric cancer

**DOI:** 10.1042/BSR20192712

**Published:** 2020-02-14

**Authors:** Galiya Yesseyeva, Batuer Aikemu, Hiju Hong, Chaoran Yu, Feng Dong, Jing Sun, Lu Zang, Minhua Zheng, Junjun Ma

**Affiliations:** 1 Department of General Surgery, Ruijin Hospital, Shanghai Jiao Tong University, School of Medicine, Shanghai 200025, P.R. China; 2 Shanghai Minimally Invasive Surgery Center, Ruijin Hospital, Shanghai Jiao Tong University, School of Medicine, Shanghai 200025, P.R. China

**Keywords:** Gastric cancer, HER2, Kaplan-Meier, Prefoldin, Prognosis

## Abstract

Prefoldin subunits (PFDN), primarily known for co-chaperone function associated with cytoskeletal rearrangement, have been found involved in epithelial–mesenchymal transition (EMT) and cancer progression. However, studies focusing on the roles of PFDN in gastric cancer (GC) remain limited. The present study aims to evaluate the prognostic values of PFDN in GC. Prognostic roles of PFDNs were analyzed via the Kaplan–Meier platform, followed by subset analysis within various clinical parameters. High mRNA expression of PFDN2, PFDN3 and PFDN4 displayed poor overall survival (OS) while PFDN5 displayed favorable OS. In HER2+ subset, PFDN2, PFDN3, PFDN4 and PFDN6 displayed poor OS. In human epidermal growth factor receptor 2 (HER2−) subset, PFDN2, PFDN3 and PFDN4 displayed poor OS. In intestinal type subset, PFDN1 and PFDN2 displayed poor OS. In diffuse-type subset, PFDN2 and PFDN6 displayed poor OS. In moderate differentiation type subset, PFDN1 displayed poor OS. In poor differentiation type subset, PFDN2 and PFDN6 displayed poor OS. In metastasis negative subset, PFDN1, PFDN2 and PFDN6 displayed poor OS. In lymph node (LN) positive subset, PFDN2 and PFDN5 displayed poor OS. The present study provided insightful clues into the poor prognostic values of PFDNs in GC patients.

## Background

Gastric cancer (GC) remains one of the major death-causing malignancies in East Asia, East Europe and South America with resilient incidence and mortality [1–5]. Although updated surgical interventions, chemotherapy and targeted drugs have enabled great progress during the last decade, the general clinical outcomes remain largely unsatisfactory across the world [6–10]. Novel prognostic indicators are in need to stratify potential risk subsets in clinical practice for GC.

Prefoldin subunits (PFDN), comprising six different subunits (PFDN1–6), is a hexameric co-chaperone complex responsible for protein-delivering to the class II eukaryotic cytosolic chaperonin c-cpn [11]. Previous studies have shown that c-cpn is required for the biogenesis of cytoskeletal-related proteins [12,13]. PFDN are primarily known for cytoskeletal assembly during the folding of actin and tubulin monomers [14]. Depletion of PFDN leads to the disruption of neuroblast polarity and overgrowth [15]. Intriguingly, our previous study demonstrates that the expression of PFDN1 positively correlates with tumor size and invasion. Meanwhile, silencing PFDN1 also exhibits inhibitory effect upon tumor proliferation and motility due to G_2_/M cell cycle dysfunction [16]. Moreover, overexpression of PFDN1 induces the tumor growth, metastasis, cellular invasion and epithelial–mesenchymal transition (EMT) of lung cancer [14]. PFDN2, via the interaction with hepatitis C virus F protein, perturbs the organization of tubulin cytoskeleton [17]. PFDN3, also known as von Hippel-Lindau (VHL) binding protein 1 (VBP1), regulates the tubulin stability by cooperating with VHL, a tumor suppressor, in *Drosophila* [18]. Remarkably, high expression of PFDN4 indicates favorable prognosis. Inhibition of PFDN4 increases the cell growth and invasiveness [19]. PFDN5 is required for the development of central nervous system and male fertility [20]. Missense mutation of PFDN5 leads to reduced function of microtubules and microfilaments, resulting in aforementioned phenotypes [20]. PFDN6 demonstrates potential diagnostic and prognostic values in childhood acute lymphoblastic leukemia (ALL) resistant to dexamethasone [21]. Nonetheless, the prognostic values of PFDN in tumor remain largely elusive, particularly in GC.

Given the close association among each PFDN member in protein interaction network, this study focuses on the prognostic values of all PFDN members in GC using web-based bioinformatics tool.

## Methods

### Protein–protein interaction networks of PFDN family members

The interaction networks of PFDN members were determined by the Search Tool for the Retrieval of Interacting Genes/Proteins (STRING, http://www.string-db.org/) [22].

### Survival analysis in Kaplan–Meier plotter

The overall survival (OS) of mRNA expression of each PFDN family members, PFDN1 (Affymetrix IDs: 201507_at), PFDN2 (Affymetrix IDs: 218336_at), PFDN3 (Synonyms:VBP1) (Affymetrix IDs: 201472_at), PFDN4 (Affymetrix IDs: 205362_s_at), PFDN5 (Affymetrix IDs: 210908_s_at), PFDN6 (Affymetrix IDs: 233588_x_at), to OS were analyzed based on Kaplan–Meier (KM) plotter (http://kmplot.com/analysis/index.php?p=service&cancer=gastric). This online tool provides a comprehensive clinical resource for prognostic analysis across several types of cancer, including GC, breast cancer, lung cancer and ovarian cancer [23]. The datasets used for GC in this study include GSE14210, GSE15459, GSE22377, GSE29272, GSE51105 and GSE62254. However, GSE62254 was excluded due to incompatible prognostic feature and potential confounding bias factors. Survival outcomes were retrieved in several subsets, including human epidermal growth factor receptor 2 (HER2), Lauren classification, histological differentiation, pathological metastasis (M) and lymph node (LN) status. Of note, mixed type (Lauren classification), well differentiation (histological differentiation), M1 and LN negative were excluded due to insufficient cases. The results were presented with hazard ratio (HR), 95% confidence intervals (95% CI) and log-rank *P*-value. *P-*value <0.05 was considered as statistically significant.

### Materials

Tumor specimens included in the present study were patients who received diagnoses of GC, who had gastrectomy surgery at Ruijin Hospital between December 2017 and September 2018. The demographic characteristics of the patients, including age, sex, body mass index (BMI), tumor size, tumor location, and the American Society of Anesthesiologists (ASA) score, were recorded. And the 7th edition of the American Joint Committee on Cancer’s guidelines was utilized for the TNM staging of GC in these patients (Table 1). Patients who had previous chemotherapy or radiotherapy were excluded.

**Table 1:** Demographics and clinicopathological characteristics

Characteristics	Counts (%)
Age (years)	
>60	*n*=27 (71.1%)
<60	*n*=11 (28.9%)
Gender	
Male	*n*=27 (71.1%)
Female	*n*=11 (28.9%)
BMI (kg/m^2^)	22.7 ± 3.1
Tobacco use, *n* (%)	
No	*n*=25 (65.8%)
Yes	*n*=13 (34.2%)
ASA score, *n* (%)	
I	*n*=14 (36.8%)
II	*n*=21 (55.3%)
III	*n*=3 (7.9%)
Tumor location	
Lower third	*n*=17 (44.7%)
Middle third	*n*=8 (21.1%)
Upper third	*n*=13 (34.2%)
Tumor size	
<4 cm	*n*=9 (23.7%)
>4 cm	*n*=29 (76.3%)
Histology	
Adenocarcinoma	*n*=8 (21%)
Mucinous	*n*=6 (15.8%)
Poorly cohesive	*n*=16 (42.2%)
Signet ring cell	*n*=8 (21%)
Tumor stage	
I	*n*=2 (5.3%)
II	*n*=5 (13.1%)
III	*n*=31 (81.6%)

*ASA; BMI; *P*-value.

### Validation of PFDN family expression in Ruijin GC cohort

Surgical specimens of formalin-fixed tissues from 38 GC patients were used for quantification of the PFDN family. Fresh tumor and paired normal tissues were frozen in liquid nitrogen and stored at −80°C. Our study protocol has been approved by the Ethics Committee of Ruijin Hospital, Shanghai Jiao Tong University, School of Medicine. Informed consent was obtained from each enrolled patient. Total RNA was extracted using TRIzol reagent (Ambion) and underwent reverse transcription reaction to generate cDNA by using Vazyme Biotech Co (Nanjing, China). PCR was conducted using the SYBR Green Master Mix (Applied Biosystems, MA, U.S.A.) and the Applied Biosystems 7900HT sequence detection system. After the reactions were complete, relative gene expression level was calculated using the 2^−ΔΔ*C*_t_^ method. The primers used for PCR are listed in Table 2. Clinical information including age, sex, stage, pathological diagnosis, TNM status and OS time were also collected for survival analysis.

**Table 2:** Quantitative real-time polymerase chain reaction primers

Name	Sequence
GAPDH Forward primer:	GGAGCGAGATCCCTCCAAAAT;
Reverse primer:	GGCTGTTGTCATACTTCTCATGG
PFDN1 Forward primer:	CGCAGACATACAGATTGAACAGC;
Reverse primer:	GAATTGCTTCCTTGGACTGAAGA.
PFDN2 Forward primer:	ATGAGCACAGCCTAGTGATCG;
Reverse primer:	ACTCCTCCAACCATGCGGTA
PFDN3 Forward primer:	AGTCCACCAACTCAATGGAGA;
Reverse primer:	CAAGCATTACATTAGCCCCCAA
PFDN4 Forward primer:	AGTGGAATCAATTCAGCGAGTG;
Reverse primer:	GCTTCAAGGTTTATGTTGCTCCC
PFDN5 Forward primer:	GCTGGACCAGATGTATGTCCC;
Reverse primer:	ATCATTTCCATGACGGCCTGT

## Results

### Prognostic values of PFDN members in GC

**Figure 1:**
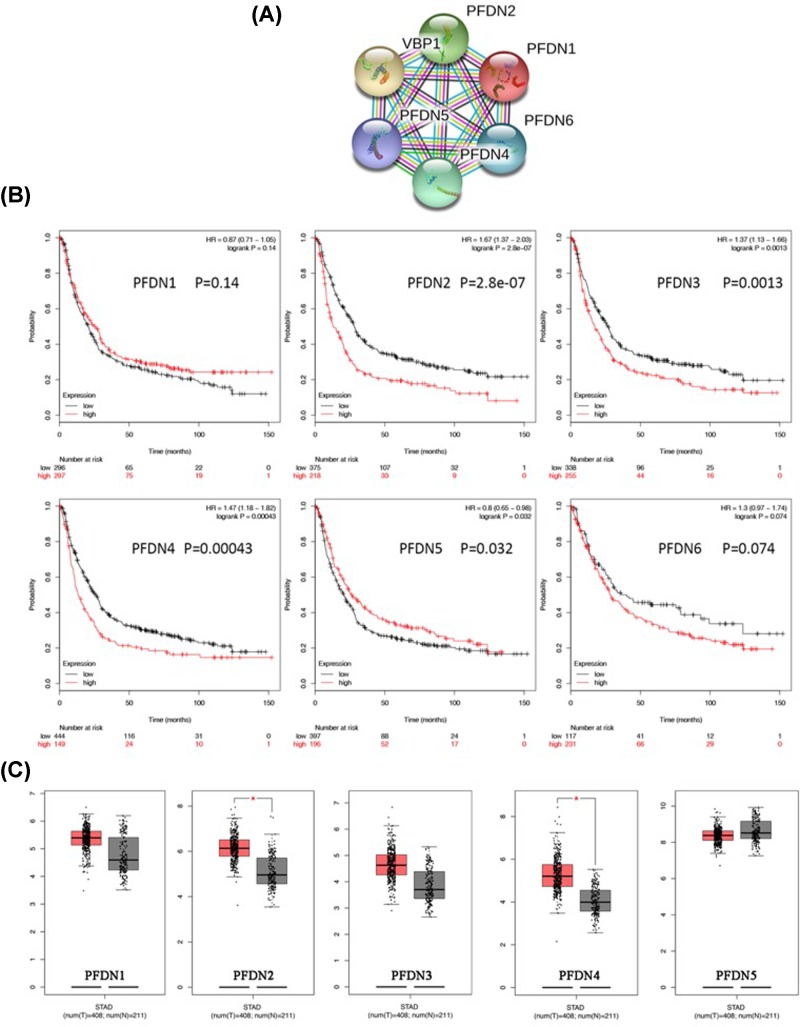
The PPI network of PFDN family (synonyms of PFDN3: VBP1) and the prognostic values of PFDN family in KM plotter (**A**) The PPI network of PFDN members; (**B**) survival curves of PFDN1 (Affymetrix IDs: 201507_at), PFDN2 (Affymetrix IDs: 218336_at), PFDN3 (VBP1, Affymetrix IDs: 201472_at), PFDN4 (Affymetrix IDs: 205362_s_at), PFDN5 (Affymetrix IDs: 210908_s_at), PFDN6 (Affymetrix IDs: 233588_x_at) for all GC patients. Red: high mRNA expression; black: low mRNA expression. (**C**) The expression levels of PFDN members in TCGA stomach adenocarcinoma (STAD) dataset.

Given the close association among each PFDN member in protein–protein interaction (PPI) network (Figure 1), it is insightful to characterize the prognostic values of PFDN members in GC systematically. Of note, in general GC group, high mRNA expression of PFDN2 (HR = 1.67, 95% CI: 1.37–2.03, *P*=2.8e-07), PFDN3 (HR = 1.37, 95% CI: 1.13–1.66, *P*=0.0013), PFDN4 (HR = 1.47, 95% CI: 1.18–1.82, *P*=0.00043) displayed significant shorter OS while PFDN5 (HR = 0.8, 95% CI: 0.65–0.98, *P*=0.032) displayed favorable outcome (Figure 1B). The results indicated that PFDN2, PFDN3 and PFDN4 might have acted as oncogenes in GC, whereas PFDN5 represented as tumor suppressor genes. Consistently, PFDN2, PFDN3 and PFDN4 showed higher expression levels, while PFDN5 showed lower levers in TCGA GC tissues (Figure 1C).

### Prognostic values of PFDN members in HER2^+/–^ subsets of GC

**Figure 2:**
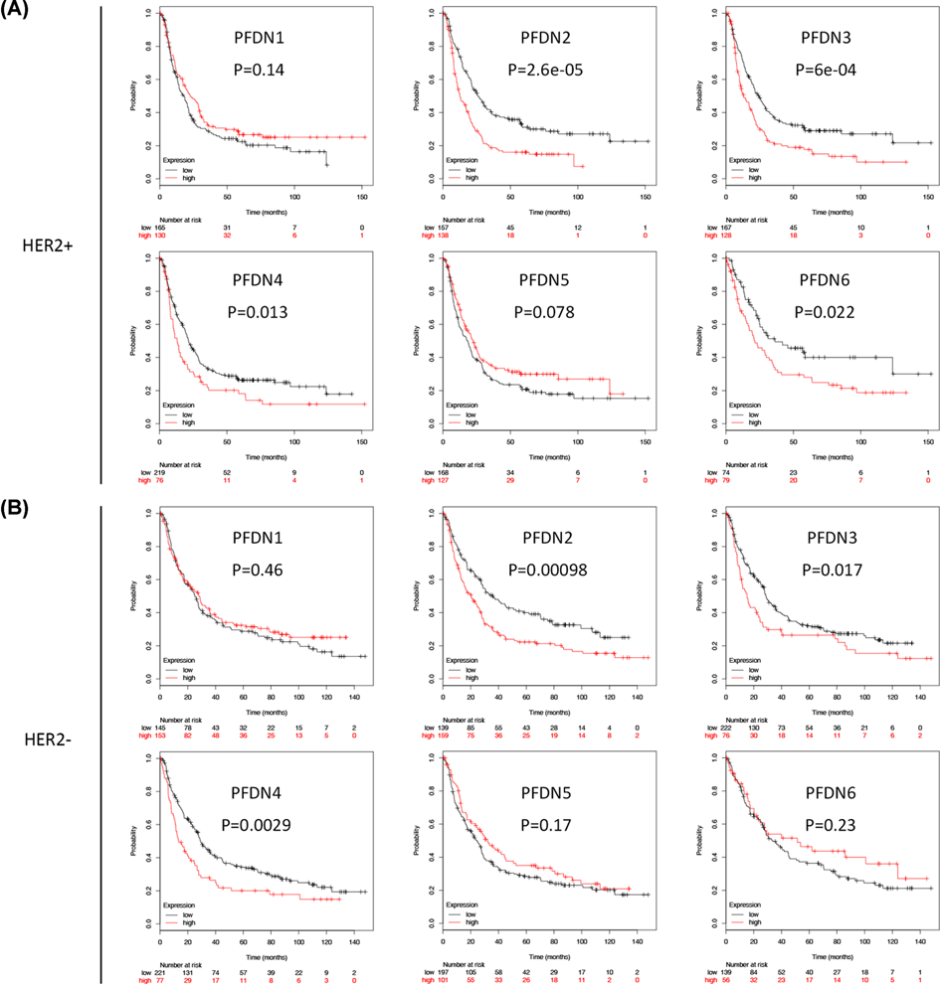
Prognostic values of PFDN members in HER2 subsets GC (**A**) Survival curves of PFDN1 (Affymetrix IDs: 201507_at), PFDN2 (Affymetrix IDs: 218336_at), PFDN3 (VBP1, Affymetrix IDs: 201472_at), PFDN4 (Affymetrix IDs: 205362_s_at), PFDN5 (Affymetrix IDs: 210908_s_at), PFDN6 (Affymetrix IDs: 233588_x_at) in HER2+ subset; (**B**) survival curves of PFDN1 (Affymetrix IDs: 201507_at), PFDN2 (Affymetrix IDs: 218336_at), PFDN3 (VBP1, Affymetrix IDs: 201472_at), PFDN4 (Affymetrix IDs: 205362_s_at), PFDN5 (Affymetrix IDs: 210908_s_at), PFDN6 (Affymetrix IDs: 233588_x_at) in HER2– subset. Red: high mRNA expression; black: low mRNA expression.

In HER2+ subset, PFDN2 (HR = 1.78, 95% CI: 1.36–2.34, *P*=2.6e-05), PFDN3 (HR = 1.6, 95% CI: 1.22–2.11, *P*=6e-04), PFDN4 (HR = 1.46, 95% CI: 1.08–1.98, *P*=0.013) and PFDN6 (HR = 1.62, 95% CI: 1.07–2.47, *P*=0.022) displayed consistently poor OS (Figure 2A). In HER2– subset, PFDN2 (HR = 1.59, 95% CI: 1.2–2.09, *P*=0.00098), PFDN3 (HR = 1.44, 95% CI: 1.06–1.96, *P*=0.017) and PFDN4 (HR = 1.57, 95% CI: 1.16–2.13, *P*=0.0029) displayed consistently poor OS (Figure 2B).

### Prognostic values of PFDN members in Lauren classification subsets of GC (intestinal and diffuse types)

**Figure 3:**
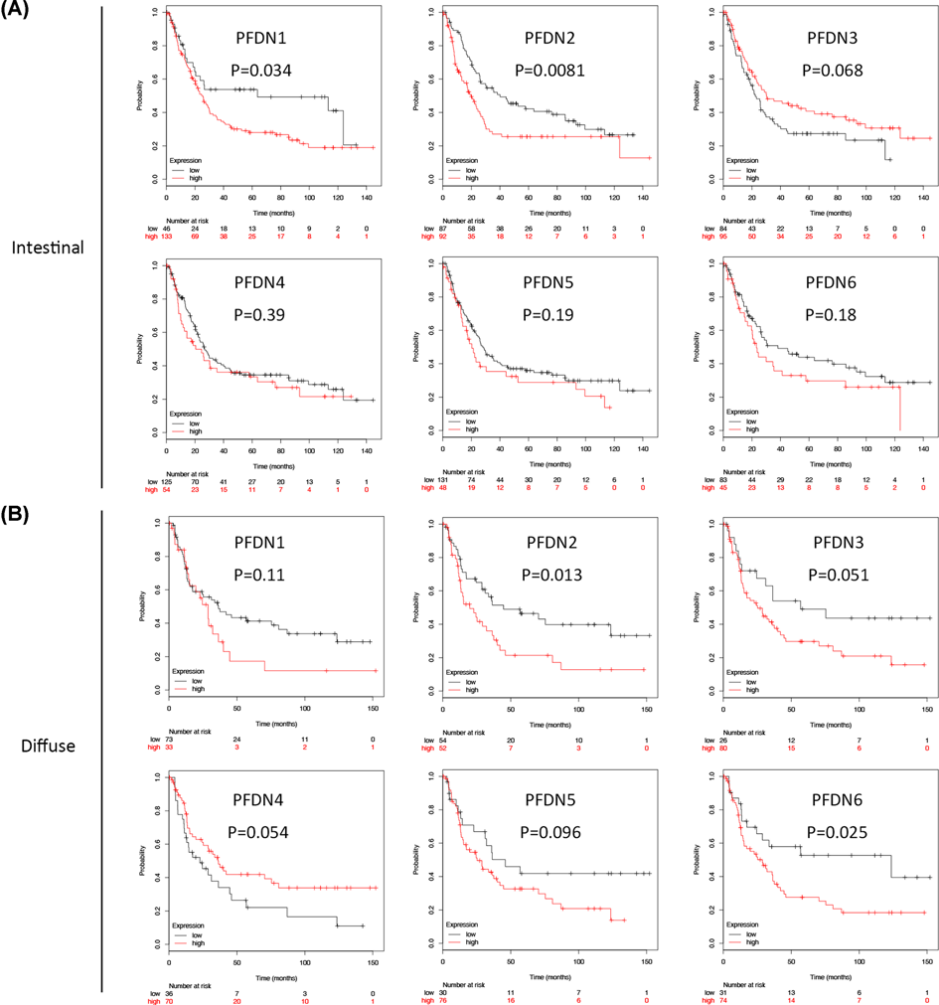
Prognostic values of PFDN members in Lauren-classification subsets GC (excluding mixed type) (**A**) Survival curves of PFDN1 (Affymetrix IDs: 201507_at), PFDN2 (Affymetrix IDs: 218336_at), PFDN3 (VBP1, Affymetrix IDs: 201472_at), PFDN4 (Affymetrix IDs: 205362_s_at), PFDN5 (Affymetrix IDs: 210908_s_at), PFDN6 (Affymetrix IDs: 233588_x_at) in intestinal subset; (**B**) survival curves of PFDN1 (Affymetrix IDs: 201507_at), PFDN2 (Affymetrix IDs: 218336_at), PFDN3 (VBP1, Affymetrix IDs: 201472_at), PFDN4 (Affymetrix IDs: 205362_s_at), PFDN5 (Affymetrix IDs: 210908_s_at), PFDN6 (Affymetrix IDs: 233588_x_at) in diffuse subset. Red: high mRNA expression; black: low mRNA expression.

In intestinal type subset, PFDN1 (HR = 1.67, 95% CI: 1.03–2.68, *P*=0.034) and PFDN2 (HR = 1.65, 95% CI: 1.14–2.41, *P*=0.0081) displayed poor OS (Figure 3A). In diffuse type subset, PFDN2 (HR = 1.86, 95% CI: 1.13–3.06, *P*=0.013) and PFDN6 (HR = 1.95, 95% CI: 1.07–3.54, *P*=0.025) displayed poor OS (Figure 3B).

### Prognostic values of PFDN members in histological-differentiation subsets of GC (moderate and poor types)

**Figure 4:**
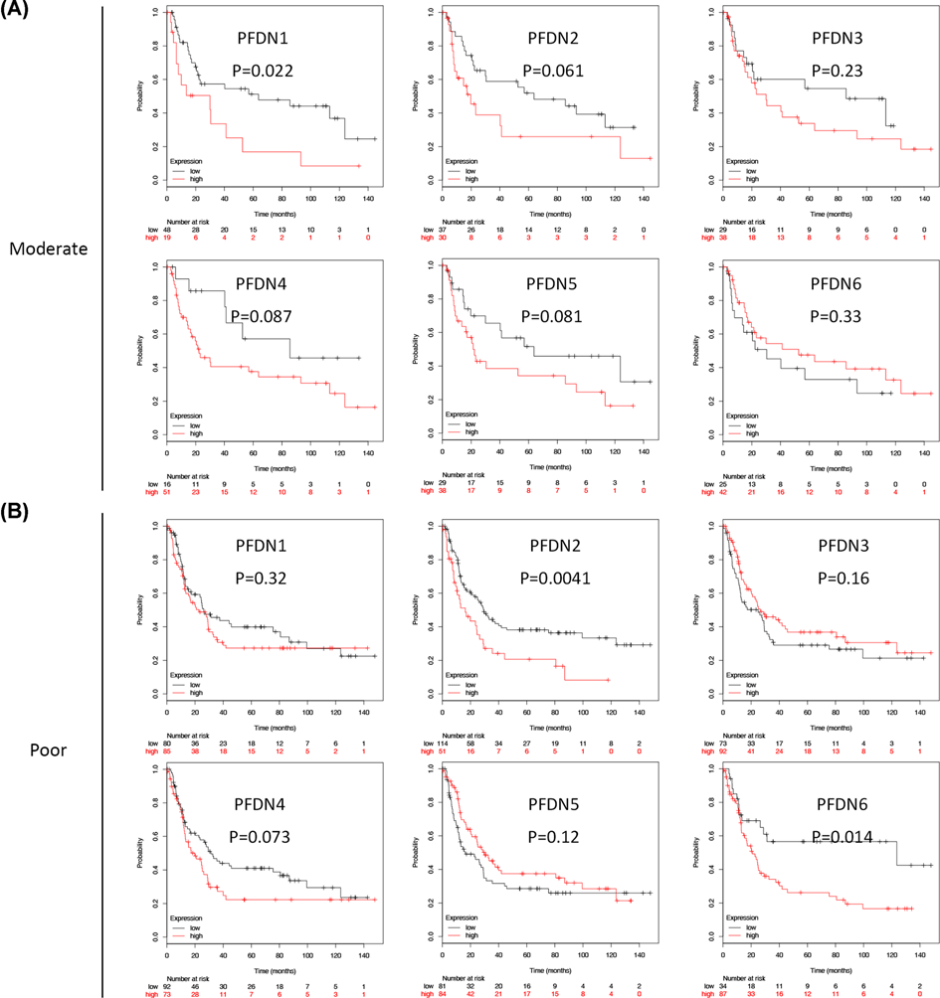
Prognostic values of PFDN members in histological differentiation subsets GC (excluding well-differentiated type) (**A**) Survival curves of PFDN1 (Affymetrix IDs: 201507_at), PFDN2 (Affymetrix IDs: 218336_at), PFDN3 (VBP1, Affymetrix IDs: 201472_at), PFDN4 (Affymetrix IDs: 205362_s_at), PFDN5 (Affymetrix IDs: 210908_s_at), PFDN6 (Affymetrix IDs: 233588_x_at) in moderate subset; (**B**) survival curves of PFDN1 (Affymetrix IDs: 201507_at), PFDN2 (Affymetrix IDs: 218336_at), PFDN3 (VBP1, Affymetrix IDs: 201472_at), PFDN4 (Affymetrix IDs: 205362_s_at), PFDN5 (Affymetrix IDs: 210908_s_at), PFDN6 (Affymetrix IDs: 233588_x_at) in poor subset. Red: high mRNA expression; black: low mRNA expression.

In moderate differentiation type subset, PFDN1 (HR = 2.18, 95% CI: 1.1–4.29, *P*=0.022) displayed poor OS (Figure 4A). In poor differentiation type subset, PFDN2 (HR = 1.83, 95% CI: 1.2–2.78, *P*=0.0041) and PFDN6 (HR = 2.07, 95% CI: 1.14–3.73, *P*=0.014) displayed poor OS (Figure 4B).

### Prognostic values of PFDN members in GC with LNs positive (LN+) and metastasis negative subsets (M0)

**Figure 5:**
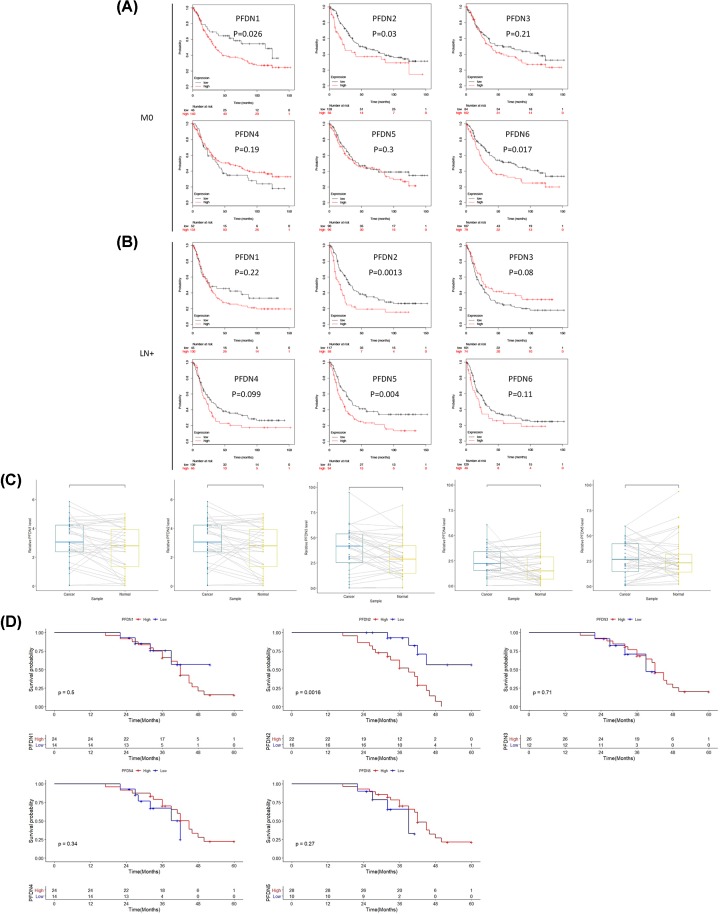
Prognostic values of PFDN members in metastasis negative (M0) and LN positive (LN+) subsets GC (**A**) Survival curves of PFDN1 (Affymetrix IDs: 201507_at), PFDN2 (Affymetrix IDs: 218336_at), PFDN3 (VBP1, Affymetrix IDs: 201472_at), PFDN4 (Affymetrix IDs: 205362_s_at), PFDN5 (Affymetrix IDs: 210908_s_at), PFDN6 (Affymetrix IDs: 233588_x_at) in M0 subset; (**B**) survival curves of PFDN1 (Affymetrix IDs: 201507_at), PFDN2 (Affymetrix IDs: 218336_at), PFDN3 (VBP1, Affymetrix IDs: 201472_at), PFDN4 (Affymetrix IDs: 205362_s_at), PFDN5 (Affymetrix IDs: 210908_s_at), PFDN6 (Affymetrix IDs: 233588_x_at) in LN+ subset. Red: high mRNA expression; black: low mRNA expression. (**C**) The expression levels of PFDN members in GC patients from Ruijin cohort. (**D**) Survival analysis of PFDN members in Ruijin cohort. *P*-values were as indicated.

In M0 type subset, PFDN1 (HR = 1.73, 95% CI: 1.06–2.82, *P*=0.026), PFDN2 (HR = 1.58, 95% CI: 1.04–2.4, *P*=0.03) and PFDN6 (HR = 1.59, 95% CI: 1.08–2.33, *P*=0.017) displayed poor OS (Figure 5A). In LN+ type subset, PFDN2 (HR = 1.88, 95% CI: 1.27–2.78, *P*=0.0013) and PFDN5 (HR = 1.73, 95% CI: 1.19–2.52, *P*=0.004) displayed poor OS (Figure 5B).

### Prognostic value of PFDN family members in GC patients from Ruijin cohort

The above results showed that PFDN family represents promising survival biomarkers of GC. To further validate the results of bioinformatics analysis, we enrolled 38 GC patients who received radical surgery in our center, the demographic and clinical characters were listed in Table 1. Moreover, the expression levels as well as the prognostic value of PFDN family were measured by quantitively PCR (qPCR) and Kaplan–Meier analysis. PCR analysis demonstrated that in comparison with normal counterparts (Figure 5C), high levels of expression of PFDN1, PFDN2, PFDN3 and PFDN4 was seen overexpressed in GC cases, while PFDN5 showed lower levels of expression in GC tissues, which was mostly consistent with bioinformatics analysis. Moreover, PFDN2 indicated worse OS of GC patents while other PFDN members did not show significant difference in patient survival (Figure 5D). We speculated that this might have been due to the relatively small sample size and needed to be further validated in prospective trails.

## Discussion

Insightfully, the present study highlighted the close association among each PFDN member, however, the intrinsic mechanism beneath remains to be fully disclosed (Figure 1A). To our knowledge, the present study was the first to comprehensively assess the prognostic roles of PFDNs using web-based tools with large samples. In the present study, PFDN members displayed significant poor OS both in general group and subset analysis, except PFDN5 in general group.

Previous studies demonstrated that members of PFDNs had been primarily involved in cytoskeletal rearrangement and developmental biology [14,15]. Meanwhile, PFDNs also were identified as interacting partners with histone deacetylase 1 protein complexes in liver cancer [24]. However, the best properties of each gene had not been fully recognized with the corresponding applications yet to be justified. The association between PFDNs and tumors could change our knowledge of PFDNs, further extending their potential prognostic roles to cancer research and clinical practice. These indications thus paved the way for subsequent comprehensively clinical investigation.

PFDN1 is constitutively involved with cytoskeletal rearrangement. Depletion of PFDN1 was associated with cytoskeletal abnormalities [25]. Marked increase in PFDN1 was noticed during the EMT process in human lung tumor [14]. Overexpression of PFDN1 was associated with increased EMT and invasiveness while knockdown showing opposite phenotypes. In fact, PFDN1 directly suppressed cyclin A by targeting the promoter of cyclin A [14]. Consistently, our previous report also found out that silencing PFDN1 could display inhibitory effects upon both proliferation and motility via G_2_/M cell cycle pathway. Moreover, the expression of PFDN1 was positively associated with tumor size and invasion in colorectal cancer (CRC) [16]. In this study, the potential prognostic role of PFDN1 has been identified in intestinal type, moderate differentiation and M0 subsets.

PFDN2, another PFDN subunit, had been previously found regulating the cytoskeleton organization [17]. Noteworthy, in this study, PFDN2 was the only PFDN subunit widely identified as prognostic indicator in both general group and subset analysis. Reasonably to presume, PFDN2 could be used as prognostic marker for GC in several subsets. However, the multivariate cox of PFDN member in the present study remains unavailable.

PFDN3 (VBP1) was previously found closely associated with the tubulin stability by cooperating with VHL [18]. It was also involved in the proteasome and autophage-mediated degradation of human MutS family protein hMSH4 [26]. The prognostic value of PFDN3 has been identified in both HER2+ and HER2− negative subsets, instead of Lauren classification and histological differentiation.

PFDN4 had been found as predictive biomarker for prognosis in CRC [19]. PFDN5 has been involved in developmental biology [20]. PFDN6 demonstrates distinct predictive values in ALL resistant to dexamethasone [21]. However, the studies of PFDN4-6 remain limited. Our result indicated that PFDN4 was a poor prognostic indicator in general GC and HER2 subsets. Interestingly, PFDN5 was identified as significantly associated with favorable OS in general GC, but failure to be a prognostic indicator in subsets analysis, including HER2, Lauren classification, histological differentiation and metastasis negative groups. However, PFDN5 was a significant poor prognostic indicator in LN+ subset analysis, contradictory to its value in general GC group. The limited cases in each subset may serve as a confounding factor to the determination of prognostic value of PFDN5.

Furthermore, we validated the results of bioinformatics analysis in our cohort which including 38 GC patients. The results of the quantitative PCR and survival analysis confirmed that PFDN2 was a potent oncogene and a promising biomarker in GC. Nonetheless, the limitation of the present study was the lack of mechanistic investigations which should be clarified in future.

## Conclusion

The present study provided insightful clues into the poor prognostic values of PFDNs in GC patients.

## Data Availability

The datasets supporting the conclusion of this article are included within the article.

## Abbreviations

ALL: acute lymphoblastic leukemia
CI: confidence interval
CRC: colorectal cancer
EMT: epithelial–mesenchymal transition
GC: gastric cancer
HER2: human epidermal growth factor receptor 2
HR: hazard ratio
LN: lymph node
OS: overall survival
PFDN: Prefoldin subunit
VBP1: von Hippel-Lindau binding protein 1
VHL: von Hippel-Lindau
